# Molecular Evaluation of RNA-Based Immune Checkpoint Profiling in Clear Cell Renal Cell Carcinoma

**DOI:** 10.3390/jcm15114311

**Published:** 2026-06-02

**Authors:** Erica Giacobbi, Valeria Palumbo, Francesca Servadei, Rita Bonfiglio, Giusella Maria Francesca Moscato, Marco Carilli, Matteo Vittori, Valerio Iacovelli, Pierluigi Bove, Alessandro Mauriello, Manuel Scimeca

**Affiliations:** 1 Department of Experimental Medicine, Torvergata Oncoscience Research (TOR), University of Rome Tor Vergata, 00133 Rome, Italy; erica.giacobbi@uniroma2.it (E.G.); valeria.palumbo@uniroma2.it (V.P.); francesca.servadei@uniroma2.it (F.S.); rita.bonfiglio@uniroma2.it (R.B.); manuel.scimeca@uniroma2.it (M.S.); 2 Department of Systems Medicine, University of Rome Tor Vergata, 00133 Rome, Italy; gius_va@virgilio.it; 3 Urology Unit, Department of Surgery, University of Rome Tor Vergata, 00133 Rome, Italy; carillimarco@gmail.com (M.C.); vittori.matteo@gmail.com (M.V.); valerio.iacovelli85@gmail.com (V.I.);

**Keywords:** immune checkpoints, clear cell renal carcinoma, immunotherapies, precision medicine

## Abstract

**Background:** Clear cell renal cell carcinoma is considered an immunologically “hot” tumor, with an abundance of tumor-infiltrating immune cells. This feature has a strong effect on therapeutic outcomes, especially in immune therapies. Within this context, immune checkpoint expression plays a central role in RCC immunotherapy, establishing new standards of care for patients with advanced RCC. However, most patients do not derive the maximum benefit from immune checkpoint inhibitors and may experience severe adverse effects. This may highlight the need to better understand the complexity of immunosuppressive mechanisms within the tumor microenvironment, which involves a variety of molecules. As such, this study aims to characterize the immune checkpoint status by investigating the co-expression (RNASeq) of the main checkpoints in a cohort of 16 ccRCC. **Results:** In the investigated cohort, the evaluation of immune checkpoint molecules revealed the complexity of immune regulatory mechanisms within the tumor microenvironment. The marked heterogeneity in the expression of eight major immune checkpoint actors, namely, PD1, PDL1, CTLA4, PDL2, VISTA, TIM3, TIGIT, and LAG3, is matched by multiple co-expressed immune checkpoint molecules, making the immune checkpoint burden score. Specifically, immune-cold tumors displayed the highest degree of co-expression, together with high levels of PDL2 and VISTA. Furthermore, the expression of immune checkpoints, both individually and collectively, was positively associated with intratumoral immune cytolytic activity, highlighting the coexistence of immune activation and immune suppression as tightly interconnected processes. **Conclusions:** The data presented here supports the concept that a ccRCC microenvironment may be driven by multiple modulatory mechanisms that allow immune escape to happen. Revealing the coordination of immune checkpoint molecules and the compensatory upregulation mechanisms may support future studies exploring multi-target immunotherapeutic strategies.

## 1. Introduction

The incidence and mortality of renal cell carcinoma (RCC) have been rising across numerous countries [1]. Among RCCs, clear cell renal cell carcinoma (ccRCC) is the most prevalent subtype, representing around 70% of all RCCs. Renal cell carcinoma is considered an immunologically “hot” tumor, characterized by enhanced activation of effector T lymphocytes, B lymphocytes, macrophages, and natural killer cells [2]. Tumor-infiltrating immune cells significantly influence therapeutic outcomes and can facilitate immune evasion through the expression of ligands that inhibit immunomodulatory receptors [3].

Within this context, immune checkpoint expression plays a central role in RCC immunotherapy. The network of immune checkpoints involves complex interactions between cancer and immune cells [4,5,6]. Among the most studied molecules, Programmed Death-1 (PD-1) and Cytotoxic T-Lymphocyte-Associated Protein 4 (CTLA-4) have been successfully targeted with monoclonal antibodies, establishing new standards of care for patients with metastatic RCC. However, a substantial proportion of patients do not achieve durable clinical benefit and may experience severe adverse effects [7,8].

According to this evidence, ccRCC could represent an optimal target for immune checkpoint-based therapies. Although immune checkpoint inhibitors have improved clinical outcomes, their efficacy remains limited by primary and acquired resistance as well as inter-patient heterogeneity, highlighting the need to better understand the coexistence of multiple immunosuppressive mechanisms within the tumor microenvironment [9,10]. In this context, increasing attention is being directed toward the simultaneous targeting of multiple immune checkpoints, with emerging strategies exploring molecules such as TIGIT, LAG3 and VISTA, often in combination with PD-1/PD-L1 inhibitors [11,12].

These challenges highlight the need for molecular approaches aimed at understanding the dynamic interactions of immune checkpoint molecules within the tumor microenvironment. Specifically, this exploratory study aims to characterize the immune checkpoint landscape and co-expression patterns of major checkpoint molecules by RNASeq analysis in a clinically annotated cohort of ccRCC, with the goal of generating biologically relevant hypotheses on coordinated immune regulatory programs within the tumor microenvironment.

## 2. Materials and Methods

### 2.1. Study Design and Patient Recruitment

A total of 16 samples of ccRCC were collected for the study. Histological and immunohistochemical analysis were performed for pathological classification on FFPE tissue, while molecular investigations were obtained from fresh frozen tissues. Specifically, histological classification and pathological review were independently performed by expert pathologists according to the World Health Organization (WHO) Classification of Urinary and Male Genital Tumors (5th Edition) [1]. Tumor grading was assessed using the WHO/ISUP grading system for renal cell carcinoma. Representative tumor areas were selected on hematoxylin and eosin-stained sections prior to molecular analyses. Tumor tissue collection was performed using a standardized protocol aimed at preventing cold ischemia until freezing in liquid nitrogen, as previously reported [13]. The study protocol was approved by the Institutional Ethical Committee of the “Policlinico Tor Vergata” (reference number # 96-19, 17 July 2019). All experimental procedures were conducted in accordance with the Code of Ethics of the World Medical Association, specifically the Declaration of Helsinki.

### 2.2. Nucleic Acid Extraction and Quality Assessment

Frozen tissue samples were lysed in buffer supplemented with β-mercaptoethanol and mechanically homogenized using a BeadBug system. Total RNA was extracted using the AllPrep Universal Kit (QIAGEN, Hilden, Germany). RNA concentration was measured by Qubit fluorometry (RNA BR assays), while RNA quality was assessed using an Agilent TapeStation with High-Sensitivity RNA ScreenTape kits (Agilent Technologies, Santa Clara, CA, USA). Samples with RNA integrity number (RIN) ≥ 4 or DV200 ≥ 60 were considered suitable for downstream library preparation.

### 2.3. Library Preparation, RNA Sequencing and NGS Data Processing

For transcriptomic profiling, ribosomal RNA was depleted with the Ribo Zero Kit (Illumina, San Diego, CA, USA), followed by library preparation using the TruSeq Stranded Total RNA Kit (Qiagen, Hilden, Germany). Sequencing was conducted on an Illumina NovaSeq 6000 platform with 150 bp paired-end reads. RNA-seq libraries generated ≥100 million total reads, with <20% rRNA content and ≥20 million reads mapping to mRNA transcripts based on the Ensembl annotation. Ribosomal depletion targeted both nuclear and mitochondrial rRNA species.

Gene expression levels were normalized as transcripts per million (TPM) and tumor immune cell infiltration was estimated using MCPCounter algorithms [14,15]. Hot and cold immune status was identified using the package TIDE [16].

### 2.4. Bioinformatic Analysis

For the bioinformatic analysis, publicly available datasets were used. Specifically, gene expression and survival data were obtained from the TCGA Kidney Renal Clear Cell Carcinoma (KIRC) dataset using the Kaplan–Meier Plotter platform (https://kmplot.com/analysis/; accessed on 22 May 2026). Overall survival analyses were performed in 530 patients, while differential gene expression analyses were conducted comparing normal kidney tissues (n = 117) and tumor samples (n = 535).

### 2.5. Statistical Analysis

For the bioinformatic analysis, survival curves were generated using the Kaplan–Meier method, and differences between groups with high and low gene expression were evaluated using the log-rank test. Hazard ratios (HRs) with 95% confidence intervals (CIs) were calculated according to the platform’s Cox proportional hazards model. For gene expression comparisons between tumor and normal tissues, *p* values were calculated using the statistical framework implemented within the Kaplan–Meier Plotter tool.

Continuous variables were summarized as median and interquartile range (IQR). For immuno-checkpoint markers, mean, standard deviation, and coefficient of variation (CV) were additionally reported to describe inter-tumor variability. Given the limited sample size (n = 16) and the non-Gaussian distribution of several markers, including TIGIT and VISTA, all inferential analyses in the institutional cohort were performed using non-parametric methods.

For checkpoint-specific dichotomization, each marker was classified as high or low according to the cohort-specific median value. A composite immune checkpoint burden score was then calculated for each tumor as the number of markers with high expression across LAG3, CTLA4, PD1, PDL1, PDL2, TIGIT, TIM3, and VISTA.

Associations between continuous marker expression and clinicopathological variables were assessed using Spearman rank correlation, including analyses with tumor grade group, tumor size, and immune cytolytic activity. Differences between groups were evaluated using the Mann–Whitney U test, including comparisons between hot and cold tumors, stage I and stage III tumors, grade group 1 and grade group 2 tumors, and T group 1 and >1 tumors. Categorical comparisons based on high/low marker expression or on the presence of a multi-checkpoint-high phenotype (≥4 high checkpoints) were assessed using Fisher’s exact test.

To account for multiple tests, *p* values were adjusted using the Benjamini–Hochberg false discovery rate (FDR) procedure within each family of tests. Given the exploratory nature of the cohort, all statistical analyses were interpreted as hypothesis-generating.

All statistical analyses were performed using GraphPad Prism 6.0 (GraphPad Software, San Diego, CA, USA), and a two-sided *p* value < 0.05 was considered statistically significant unless otherwise specified.

## 3. Results

### 3.1. Clinical Characteristics of the Institutional Cohort

To further explore the biological relationships between immune checkpoint pathways within individual tumors, we performed a detailed molecular analysis in an independent institutional cohort of clear cell RCC.

The cohort included 16 clear cell RCC cases. Median age was 67.5 years (IQR 58.2–72.5), with a male predominance (12/16, 75.0%). Grade group 1 accounted for 11 cases and grade group 2 for five cases. Stage information was available for 13 tumors, of which eight were stage I and five were stage III. Lymph node metastases at diagnosis were observed in 2/16 cases (12.5%). Hot tumors represented 12 cases, whereas four were classified as cold. The main Clinicopathological are reported in Table 1.

Immune checkpoint expression heterogeneity in the cohort.

**Figure 1:**
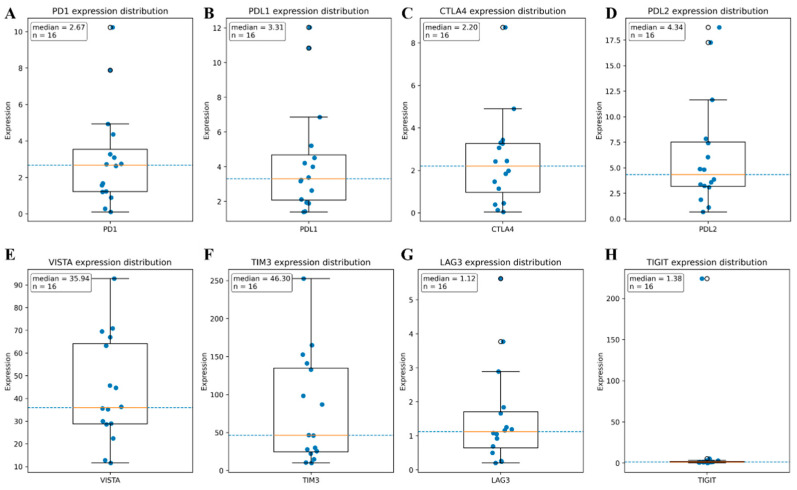
Distribution of immune checkpoint expression in the RCC cohort. Expression levels of eight immune checkpoint molecules were evaluated across the clear cell renal cell carcinoma cohort (n = 16). Boxplots show the distribution of expression values for PD1 (**A**), PDL1 (**B**), CTLA4 (**C**), PDL2 (**D**), VISTA (**E**), TIM3 (**F**), LAG3 (**G**), and TIGIT (**H**). Each dot represents an individual tumor sample. The horizontal line within each box indicates the median, the box limits represent the interquartile range (IQR), and whiskers indicate the range of observed values. The dashed horizontal line corresponds to the median expression value used as a cutoff for dichotomization into high- and low-expression groups in subsequent analyses.

Checkpoint expression was markedly heterogeneous across cases (Figure 1). The most variable markers were VISTA (median 45.17, CV 237.4%) (Figure 1E), TIM3 (median 46.30, CV 90.8%) (Figure 1F), and TIGIT (median 1.38, CV 358.2%) (Figure 1G). Across the full panel, median cutoffs used for dichotomization were 1.12 for LAG3, 2.20 for CTLA4, 2.68 for PD1, 3.31 for PDL1, 4.34 for PDL2, 1.39 for TIGIT, 46.30 for TIM3, and 45.17 for VISTA.

### 3.2. Immune Checkpoint Burden and Tumor Immune Phenotype

**Figure 2:**
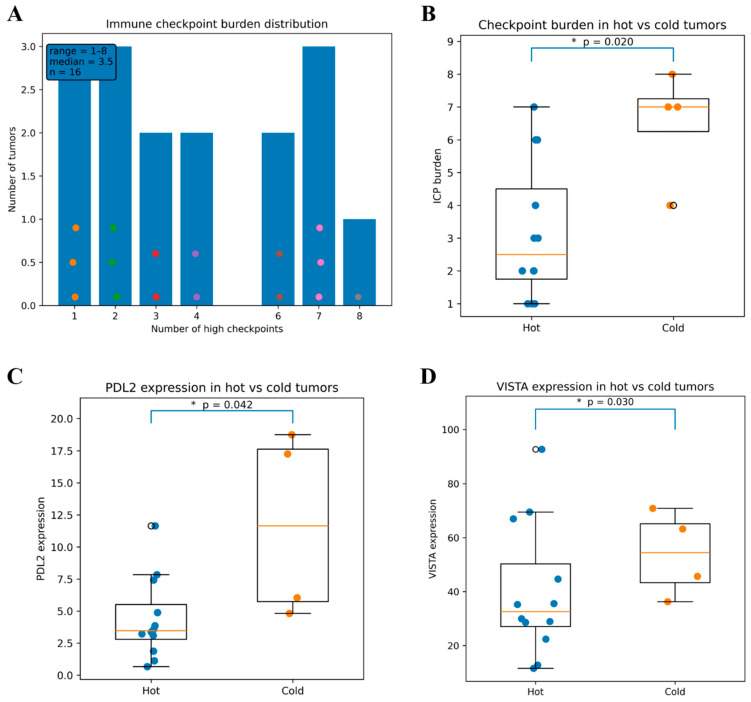
Immune checkpoint burden and association with tumor immune phenotype. (**A**) Distribution of the immune checkpoint burden across the RCC cohort. The burden score represents the number of immune checkpoints expressed above the median cutoff for each tumor. Values ranged from one to eight high checkpoints, with a median burden of 3.5. Colored dots indicate individual tumors. (**B**) Comparison of immune checkpoint burden between hot and cold tumors. Cold tumors exhibited a significantly higher checkpoint burden compared with hot tumors (median 7.0 vs. 2.5, Mann–Whitney *p* = 0.020). Each dot represents an individual tumor; boxes indicate the interquartile range and whiskers the range of observed values. Cold tumors showed higher PDL2 expression (median 11.65 vs. 3.47, Mann–Whitney *p* = 0.042) (**C**) and VISTA expression than hot tumors (median 1849.92 vs. 35.41, Mann–Whitney *p* = 0.030) (**D**).

At the patient level, the immuno-checkpoint burden score ranged from one to eight high markers. The most frequent burden states were one, two, and seven high checkpoints (three cases each), followed by three, four, and six high checkpoints (two cases each), while one tumor showed high expression for all eight checkpoints (Figure 2A). Cold tumors displayed a substantially higher burden than hot tumors (median 7.0 vs. 2.5, Mann–Whitney *p* = 0.016) (Figure 2B). This pattern was accompanied by higher PDL2 (median 11.65 vs. 3.47, *p* = 0.042) (Figure 2C) and VISTA (median 1849.92 vs. 35.41, *p* = 0.030) (Figure 2D) expression in cold tumors.

### 3.3. Association with Immune Cytolytic Activity

**Figure 3:**
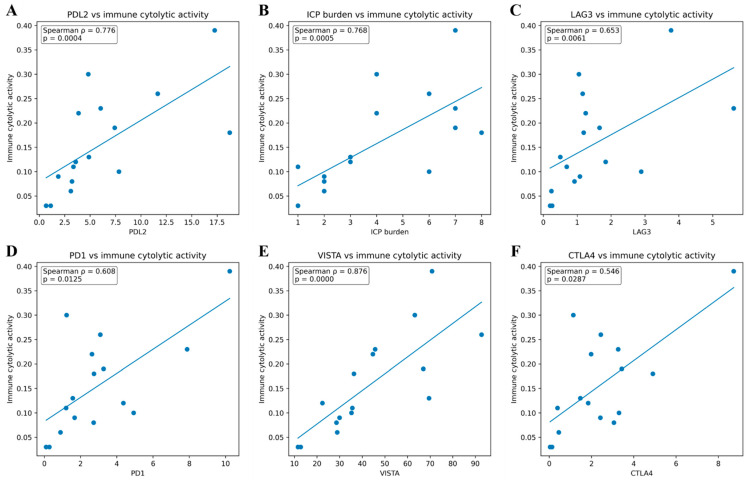
Associations between immune checkpoint expression and immune cytolytic activity in RCC. Scatter plots showing the relationship between immune checkpoint signaling and immune cytolytic activity across the RCC cohort. Each dot represents an individual tumor sample, and the blue line indicates the linear regression trend. (**A**) PDL2 expression showed the strongest association with immune cytolytic activity (Spearman ρ = 0.776, *p* = 0.0004). (**B**) Immune checkpoint burden was also positively correlated with immune cytolytic activity (ρ = 0.768, *p* = 0.0005). (**C**) LAG3 expression demonstrated a positive correlation with immune cytolytic activity (ρ = 0.653, *p* = 0.0061). (**D**) PD1 expression was similarly associated with increased cytolytic activity (ρ = 0.608, *p* = 0.0125). (**E**) VISTA expression showed a strong positive association with immune cytolytic activity (ρ = 0.876, *p* < 0.0001). (**F**) CTLA4 expression also correlated with cytolytic activity (ρ = 0.546, *p* = 0.0287).

Immune cytolytic activity showed the clearest biological associations with immune checkpoint expression (Figure 3). It correlated positively with PDL2 (Spearman ρ = 0.776, FDR = 0.0037) (Figure 3A), ICP burden (ρ = 0.745, FDR = 0.0041) (Figure 3B), LAG3 (ρ = 0.653, FDR = 0.0182) (Figure 3C), PD1 (ρ = 0.608, FDR = 0.0267) (Figure 3D), VISTA (ρ = 0.596, FDR = 0.0267) (Figure 3E), and CTLA4 (ρ = 0.546, FDR = 0.0430) (Figure 3F). No significant correlations were observed with tumor size, and no checkpoint remained significantly associated with grade group after multiple-testing correction, although TIM3 showed a non-significant inverse trend with grade.

### 3.4. Coordinated Checkpoint Programs in RCC

**Figure 4:**
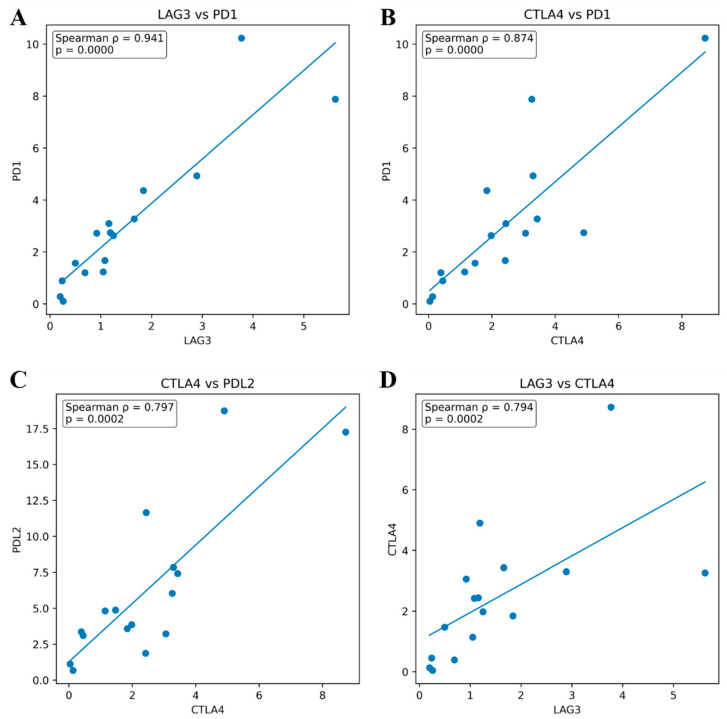
Pairwise correlations among immune checkpoint molecules in RCC. Scatter plots showing pairwise correlations between immune checkpoint expression levels across the RCC cohort. (**A**) LAG3 and PD1 expressions showed the strongest correlation across the cohort (Spearman ρ = 0.941, *p* < 0.0001). (**B**) CTLA4 and PD1 expressions were also highly correlated (ρ = 0.874, *p* < 0.0001). (**C**) CTLA4 and PDL2 expressions demonstrated a strong positive association (ρ = 0.797, *p* = 0.0002). (**D**) LAG3 and CTLA4 expressions were similarly correlated (ρ = 0.794, *p* = 0.0002).

Pairwise checkpoint analysis identified a tightly co-regulated axis involving LAG3, CTLA4, PD1, and PDL2 (Figure 4). The strongest pairwise correlations were observed for LAG3-PD1 (ρ = 0.941, FDR = 0.0000) (Figure 4A), CTLA4-PD1 (ρ = 0.874, FDR = 0.0001) (Figure 4B), CTLA4-PDL2 (ρ = 0.797, FDR = 0.0017) (Figure 4C), and LAG3-CTLA4 (ρ = 0.794, FDR = 0.0017) (Figure 4D). Collectively, these findings support the presence of coordinated checkpoint programs in a subset of RCCs, with particularly strong links to the immune cytolytic compartment.

### 3.5. Immune Checkpoint Gene Expression and Prognostic Significance in TCGA RCCs

**Figure 5:**
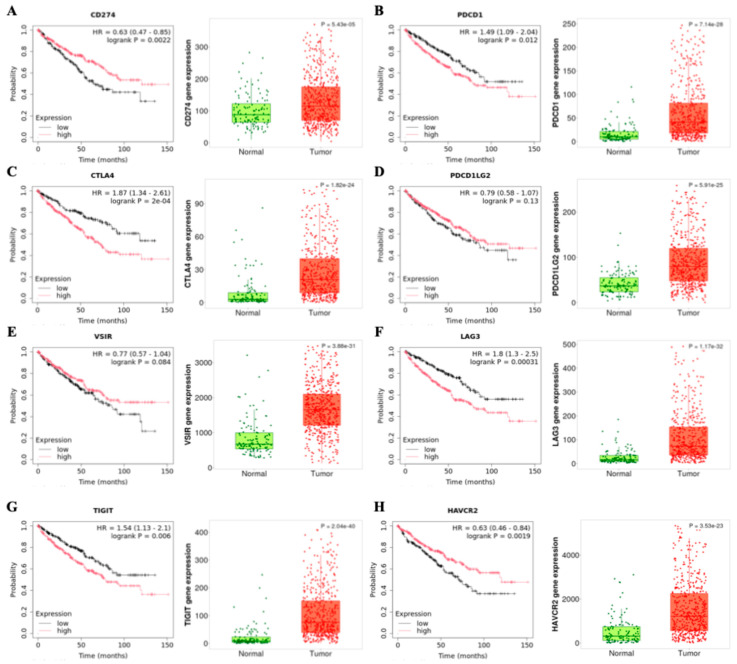
Bioinformatic analysis of immune checkpoint gene expression and prognostic impact in TCGA clear cell renal cell carcinoma. Kaplan–Meier survival analysis and differential gene expression of immune checkpoint genes were performed using the Kaplan–Meier Plotter platform for the TCGA Kidney Renal Clear Cell Carcinoma cohort. Overall survival was evaluated in 530 patients, while gene expression differences were assessed between normal kidney tissues (n = 117) and tumor samples (n = 535). For each gene, the left panel shows Kaplan–Meier survival curves comparing patients with high versus low gene expression, and the right panel shows gene expression levels in normal versus tumor tissues. (**A**) CD274 (PD-L1); (**B**) PDCD1 (PD-1); (**C**) CTLA4; (**D**) PDCD1LG2 (PD-L2); (**E**) VSIR (VISTA); (**F**) LAG3; (**G**) TIGIT; (**H**) HAVCR2 (TIM-3).

To further investigate the clinical and biological relevance of immune checkpoint pathways in clear cell RCC, we analyzed publicly available transcriptomic data from the TCGA Kidney Renal Clear Cell Carcinoma (KIRC) cohort using the Kaplan–Meier Plotter platform. Overall survival was evaluated in 530 patients, while differential gene expression analyses were performed between normal kidney tissues (n = 117) and tumor samples (n = 535) (Figure 5).

Among the investigated immune checkpoint molecules, increased expression of PDCD1 (PD-1), CTLA4, LAG3, and TIGIT was significantly associated with worse overall survival. In particular, CTLA4 (HR = 1.87, 95% CI 1.34–2.61, *p* = 2 × 10^−4^) and LAG3 (HR = 1.80, 95% CI 1.30–2.50, *p* = 3.1 × 10^−4^) showed the strongest negative prognostic impact, followed by TIGIT (HR = 1.54, 95% CI 1.13–2.10, *p* = 0.006) and PDCD1 (HR = 1.49, 95% CI 1.09–2.04, *p* = 0.012) (Figure 5B,C,F,G). In contrast, higher CD274 (PD-L1) and HAVCR2 (TIM-3) expressions were associated with more favorable survival outcomes, whereas PDCD1LG2 (PD-L2) and VSIR (VISTA) did not show significant prognostic associations.

Consistent with these findings, all investigated immune checkpoint genes were significantly upregulated in tumor tissues compared with normal kidney samples, supporting the existence of a broadly activated immunoregulatory microenvironment in ccRCC (Figure 5). Notably, the checkpoints associated with poorer prognosis, including PDCD1, CTLA4, LAG3, and TIGIT, also displayed markedly increased expression in tumor tissues relative to normal kidney, further supporting their potential biological relevance in RCC progression and immune escape mechanisms.

## 4. Discussion

In the present exploratory study, the evaluation of immune checkpoint molecules at the mRNA level revealed a marked heterogeneity in the expression of eight major immune checkpoints, namely PD1, PDL1, CTLA4, PDL2, VISTA, TIM3, TIGIT, and LAG3, supporting the existence of complex and coordinated immune regulatory programs within the ccRCC microenvironment.

Clear cell renal cell carcinoma has long been recognized as an immunogenic cancer, making it an optimal target for immune checkpoint (IC)-based therapies [17]. Although immune checkpoint inhibitors have led to substantial improvements in clinical outcomes for patients with RCC [18,19,20,21], their overall therapeutic efficacy remains limited. This is largely due to the presence of both primary and acquired resistance mechanisms, as well as significant inter-patient heterogeneity, which together contribute to variable and often suboptimal treatment responses [22,23,24,25]. These limitations underscore the need for a deeper understanding of coexisting immunosuppressive mechanisms that shape tumor behavior and influence therapeutic response to optimize patient stratification [12,23]. In this context, the study of immune checkpoint molecules is pivotal [26]. Beyond established immune checkpoint inhibitors such as those targeting the PD-1 and CTLA-4 pathways, the development of new ICIs is widening the range of targetable molecules [27]. Early-phase clinical trials are actively exploring TIGIT-targeting strategies in RCC, including combinations with PD-1/PD-L1 inhibitors and anti-angiogenic agents, LAG3-directed bispecific antibodies, or antibodies targeting VISTA [28,29,30]. These approaches reflect a growing interest in multi-checkpoint blockade strategies aimed at enhancing antitumor immunity across both treatment-naive and pre-treated settings.

Indeed, multiple checkpoints were co-expressed within individual tumors, with immune-cold tumors displaying the highest degree of co-expression, as captured by the immune checkpoint burden score. Furthermore, the expression of immune checkpoints, both individually and collectively, was positively associated with intratumoral immune cytolytic activity, highlighting the coexistence of immune activation and immune suppression as tightly interconnected processes.

Accordingly, in our cohort, we observed a great variability in the expression of immune checkpoints; in particular, VISTA, TIM3, and TIGIT. The variability of expression could be important in the stratification of patients, allowing the identification of clusters of patients which may require a personalized approach with a combination of checkpoint blockade to achieve maximal efficacy [24,31].

In fact, the modulation of the antitumor immune response is also mediated by the coordinated and context-dependent expression of various immune checkpoints. Expression of immune T-cell inhibitory molecules may also be associated with various states of T-cell tolerance and exhaustion [32,33,34]. In physiological conditions, this coordinated regulation is essential for preventing excessive activation of T-cell-mediated responses [35]. However, in a pro-tumor microenvironment, it can lead to the generation of exhausted T cells [36,37,38], which are abundant in the RCC microenvironment [38,39]. In our study emerged that in most cases the immune landscape is characterized by the expression of at least two immune checkpoints and a high ICP burden score. This pattern was particularly evident in immune-cold tumors, which showed significantly higher checkpoint burden as well as increased expression of PDL2 and VISTA, suggesting that immune exclusion or dysfunction may be sustained by the redundant activation of inhibitory signaling axes. These findings are consistent with the notion that RCC immune evasion is not merely driven by lack of immune infiltration, but rather by modulating suppression of an existing immune response.

Another phenomenon known to influence the efficacy of immunotherapy is intratumoral cytolytic activity, which is regulated by both immune checkpoint pathways and the composition of the tumor microenvironment (TME) [40,41].

Cytolytic activity resides in the ability of T lymphocytes and natural killer (NK) cells to recognize neoplastic target cells and release cytotoxic molecules such as perforin and granzymes, thereby inducing apoptosis [41]. Consequently, the presence of substantial intratumoral cytolytic activity is generally associated with a favorable prognosis, and the cytolytic activity of cytotoxic T lymphocytes within the TME represents a key determinant of response to immune checkpoint inhibitors in many malignant tumors, including renal cell carcinoma.

In this context, our findings provide further insight into the functional interplay between immune activation and immune suppression within the ccRCC microenvironment. Despite cytolytic activity being traditionally associated with effective antitumor immunity and favorable clinical outcomes, its strong positive correlation with multiple immune checkpoint molecules, including PDL2, PD1, CTLA4, LAG3, and VISTA, as well as with the overall checkpoint burden, suggests the coexistence of compensatory immunosuppressive mechanisms. This apparent paradox reflects a state of adaptive immune resistance, in which tumor-infiltrating cytotoxic lymphocytes actively produce interferon-γ and other inflammatory signals that, in turn, induce the upregulation of inhibitory checkpoints in both tumor and immune cells. Consequently, highly cytolytic tumors may also represent those with the most pronounced immune exhaustion signatures, potentially limiting the effectiveness of single-agent checkpoint blockade. Notably, these data may reflect a state of functional exhaustion of CD8^+^ T lymphocytes within the tumor microenvironment, also suggesting that, in RCC, therapeutic success with immune checkpoint inhibitors is not solely related to the presence of cytotoxic T lymphocytes but also to their functional state.

Lastly, our data support coordinated activity among checkpoint pathways commonly targeted in cancer immunotherapy, consistent with prior reports highlighting interactions among LAG-3–PD-1, LAG-3–CTLA-4, and CTLA-4–PD-1 [42]. These coordinated checkpoint patterns may provide a biological rationale for future investigation of combined immunotherapy approaches in selected ccRCC subsets.

The present study has some limitations, primarily related to the relatively small size of the institutional cohort. Consequently, the findings should be interpreted as exploratory and hypothesis-generating rather than definitive evidence of clinically generalizable immune checkpoint programs in ccRCC. Nevertheless, the adoption of conservative non-parametric statistical approaches, FDR correction, and the integration with large publicly available datasets support the biological consistency of the observed associations. Another limitation of the present study is the lack of an independent validation cohort. Although TCGA-derived analyses were used to provide external biological support, the checkpoint interaction patterns identified in the institutional cohort should be considered preliminary and require validation in larger multicenter studies. Lastly, given the exploratory nature of the study and the limited cohort size, the reported associations should be interpreted cautiously despite FDR correction, as multiple comparisons may still increase the risk of type I errors.

## 5. Conclusions

Concerning the identification of new biomarkers for precision medicine [43,44,45,46], our findings provide preliminary evidence supporting the concept that immune escape in ccRCC is driven not only by immune exclusion but also by the dynamic modulation of inhibitory pathways. The coordinated activity among checkpoint molecules, together with compensatory upregulation mechanisms, provides a strong rationale for the development of specific immunotherapeutic strategies designed to target multiple immune checkpoints simultaneously.

Therefore, a deeper characterization of immune checkpoint networks and their functional interactions may contribute to future patient stratification strategies and to the development of more personalized immunotherapeutic approaches following independent validation.

## Figures and Tables

**Table 1:** Clinicopathological characteristics of the RCC cohort.

Characteristic	n (%)/Median (IQR)
**Patients**	16
**Age, years**	67.5 (58.2–72.5)
**Male sex**	12 (75.0%)
**Grade group**	
**1**	11 (68.8%)
**2**	5 (31.2%)
**Tumor stage**	
**I**	8 (61.5%)
**III**	5 (38.5%)
**Immune phenotype**	
**Hot**	12 (75.0%)
**Cold**	4 (25.0%)
**Lymph node metastasis**	
**yes**	2 (12.5%)
**no**	14 (87.5%)

## Data Availability

All data are included in the manuscript or available from corresponding authors upon reasonable request.
